# Targeting Cyclin-Dependent Kinases in Human Cancers: From Small Molecules to Peptide Inhibitors

**DOI:** 10.3390/cancers7010179

**Published:** 2015-01-23

**Authors:** Marion Peyressatre, Camille Prével, Morgan Pellerano, May C. Morris

**Affiliations:** Institut des Biomolécules Max Mousseron, IBMM-CNRS-UMR5247, 15 Av. Charles Flahault, 34093 Montpellier, France; E-Mails: marion.peyressatre@univ-montp1.fr (M.P.); camille.prevel@univ-montp1.fr (C.P.); morgan.pellerano@univ-montp1.fr (M.P.)

**Keywords:** CDK/Cyclin Kinase, cell cycle, cancer, inhibitor, peptide, small molecule

## Abstract

Cyclin-dependent kinases (CDK/Cyclins) form a family of heterodimeric kinases that play central roles in regulation of cell cycle progression, transcription and other major biological processes including neuronal differentiation and metabolism. Constitutive or deregulated hyperactivity of these kinases due to amplification, overexpression or mutation of cyclins or CDK, contributes to proliferation of cancer cells, and aberrant activity of these kinases has been reported in a wide variety of human cancers. These kinases therefore constitute biomarkers of proliferation and attractive pharmacological targets for development of anticancer therapeutics. The structural features of several of these kinases have been elucidated and their molecular mechanisms of regulation characterized in depth, providing clues for development of drugs and inhibitors to disrupt their function. However, like most other kinases, they constitute a challenging class of therapeutic targets due to their highly conserved structural features and ATP-binding pocket. Notwithstanding, several classes of inhibitors have been discovered from natural sources, and small molecule derivatives have been synthesized through rational, structure-guided approaches or identified in high throughput screens. The larger part of these inhibitors target ATP pockets, but a growing number of peptides targeting protein/protein interfaces are being proposed, and a small number of compounds targeting allosteric sites have been reported.

## 1. Introduction

### 1.1. Cyclin-Dependent Kinases—From Cell Cycle Control to Physiological Regulation

Cyclin-dependent kinases [CDKs] were first identified independently in starfish, *Xenopus* and yeast and cloned in the 1970s–1980s as gene products involved in regulation of the cell division cycle [1,2,3,4,5,6]. These serine/threonine proline-directed kinases, which are inactive in their monomeric form, associate with a family of regulatory subunits, cyclins, named after their periodic profiles of expression and degradation, to form functional heterodimeric complexes [7,8,9]. The first CDK/Cyclin complexes to be characterized were *bona fide* regulators of cell growth and division, involved in the tight and timely control of cell cycle progression, through phosphorylation of substrates involved in DNA replication, chromatin condensation, assembly of the mitotic spindle and disassembly of the nuclear envelope. For this reason, they were thereafter considered as the “master regulators” of cell cycle progression, molecular engines that drive cell cycle transitions [10,11,12].

**Figure 1:**
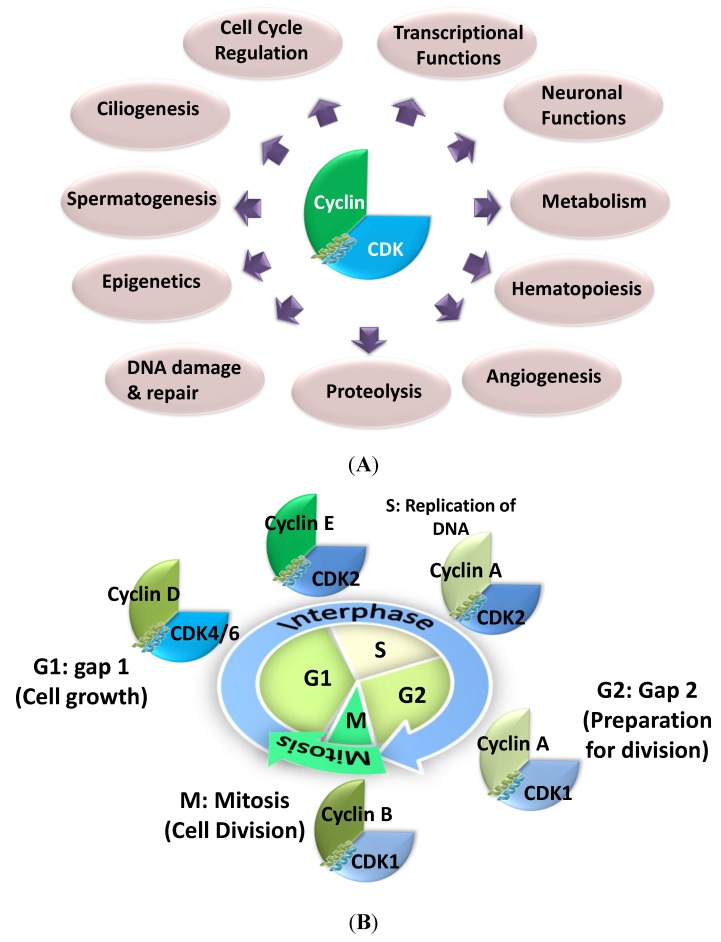
Functional Diversity of Cyclin-dependent Kinases. (**A**) Schematic representation of the functional diversity of Cyclin-dependent kinases; (**B**) Cell cycle regulation by CDK/cyclins: CDK1/cyclin B during the mitosis, CDK4 and 6/cyclin D for progression through G1 phase, CDK2/cyclin E for G1/S transition, CDK2/cyclin A at the S phase and CDK1/cyclin A for progression through G2 phase.

To date, twenty different CDKs have been reported in mammalian cells and about the same number of cyclins [13]. However, not all of them are regulators of cell cycle progression, and several of these kinases are involved in multiple functions (Figure 1A and Table 1) [14]. Indeed, more recent research has revealed the existence of specific CDK/Cyclin heterodimers whose functional implications are being uncovered in transcriptional processes and other non-cell cycle functions, as well as in pathological settings [13,14,15,16]. Hence, the functional diversity of this small group of protein kinases is important, and it is now fully recognized that CDK/Cyclins are involved in a wide variety of biological processes, including transcriptional regulation, metabolism, neuronal differentiation and development [14]. 

#### 1.1.1. *Bona Fide* Cell Cycle CDK/Cyclins

**Figure 2:**
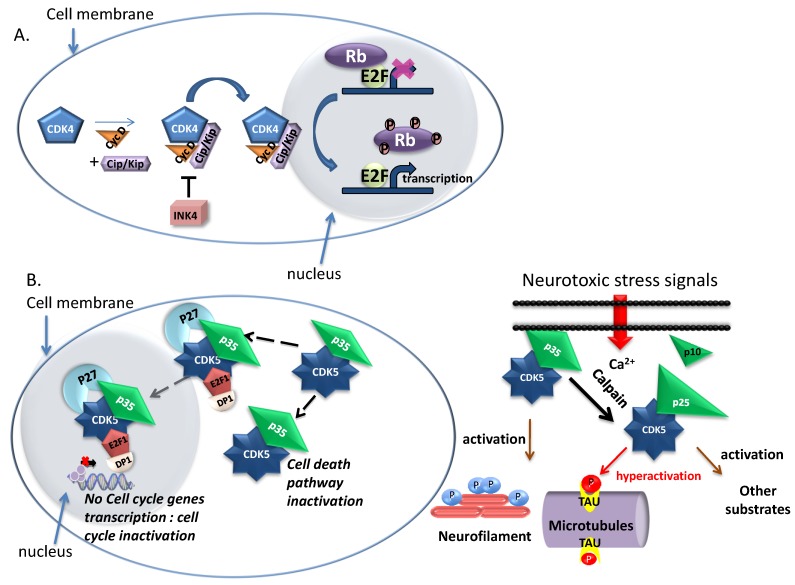
Functions of CDK4/Cyclin D and CDK5/p25. (**A**) Function and regulation of CDK4/cyclin D: The expression of cyclin D (orange) via mitogenic signals leads to its association with CDK4 (blue) and formation of the complex. The binding with Cip/Kip proteins (light purple) is required for complex assembly and its nuclear localization. The activity of CDK4/cyclin D activity is negatively regulated by interaction with the INK4 protein family (pink). Once activated, CDK4/cyclin D regulates the progression through G1 phase and the transition into S phase. CDK4/cyclin D phosphorylates the retinoblastoma protein (Rb) (dark purple) inducing the derepression of E2F transcription factors (green) which allows the transcription of genes which are required for G1/S transition; (**B**) Different functions of CDK5 in the nervous system and schematic representation of CDK5 regulation through cleavage of p35 to p25 at the cellular membrane; when CDK5/p35 binds p27 and E2F1 and DP1 in the nucleus, the cell cycle is inactivated. When p27 is degraded CDK5/p35 returns to the cytoplasm and the cell death pathway is inactivated.

CDK1, CDK2, CDK4 and CDK6 and their associated Cyclins A, B, D, E can be considered *bona fide* cell cycle regulators. Whilst these CDKs are widely, ubiquitously and constantly expressed throughout the cell cycle, their cyclin partners are periodically expressed and degraded at specific phases of the cell cycle, or in specific cells or tissues. Hence, the spatio-temporal expression profiles of cyclins regulate activities of CDKs in an orderly fashion, thereby ensuring timely cell cycle progression [17,18] **(**Figure 1B). When quiescent cells (G0 phase) are stimulated to enter the cycle by mitogenic growth factors, notably via Ras signaling pathway, expression of D-type cyclins promotes progression through G1 phase, through association and activation of CDK4 and CDK6, thereby promoting phosphorylation of Retinoblastoma pocket protein family members (p107, p130, pRb) [19]. Phosphorylation of Rb members partially inactivates their function as transcriptional repressors [20], leading to derepression of E2F transcription factors and consequent expression of genes which are required for G1/S transition, including Cyclin E in late G1 (Figure 2A). This in turn enables activation of CDK2/cyclin E, which further phosphorylates Rb, thereby promoting complete release of E2F factors, inducing their maximal action as transcriptional activators and facilitating progression through G1 [21,22].

**Table 1:** Functions of CDK/Cyclins.

CDK	In complex with	Cell cycle function	Transcriptional function	Other functions	References
CDK1	Cyclin B	mitosis	+	stem cell self-renewal	[23,24,25,26,27,28,29,30,31,32,33,34,35]
				DNA damage repair	
				epigenetic regulation	
CDK2	Cyclin E	G1/S transition	+	stem cell self-renewal	[26,28,31,35,36,37,38]
	Cyclin A	S phase		epigenetic regulation	
CDK3	Cyclin C	G1 phase	+	DNA damage repair	[39,40,41]
CDK4	Cyclin D	G1 phase		epigenetic regulation	[19,36,42]
CDK5	p35		+	neuronal functions	[35,43,44,45,46,47,48,49]
				epigenetic regulation	
				glycogen synthesis	
				insulin secretion	
CDK6	Cyclin D	G1 phase			[15,36]
CDK7	Cyclin H	CDK-activating	+		[50]
CDK8	Cyclin C	regulator of multiple steps	+	Wnt/β-catenin signaling	[51,52,53,54]
				inhibition of lipogenesis	
CDK9	Cyclin T, K		+ (cyclin T)	DNA damage repair (cyclin K)	[55,56]
CDK10	Cyclin M	G2/M transition	+		[57,58,59]
CDK11	Cyclin L			splicing regulation	[60]
CDK12	Cyclin K, L		+ (cyclin K)	splicing regulation (cyclin L)	[61,62,63,64]
				DNA damage repair (cyclin K)	
CDK13	Cyclin K, L			splicing regulation (cyclin L)	[65]
CDK14	Cyclin Y			Wnt/β-catenin signaling	[66]
CDK15					
CDK16	Cyclin Y			trafficking of synaptic proteins and synapse remodeling	[67,68,69]
				spermatogenesis	

CDK2/Cyclin E coordinates entry into S phase through additional phosphorylation of pocket protein members, thereby completely inactivating their function, and further promotes initiation of DNA synthesis, replication and progression through S phase together with CDK2/Cyclin A. Morever, several studies show that CDK2/Cyclin A primes and coordinates activation CDK1/Cylin B at the centrosome and in the nucleus during G2 phase. At a later stage, Cyclin A associates with CDK1 and is finally replaced by Cyclin B to coordinate entry and progression through mitosis [9,10,11,12,22,70].

#### 1.1.2. Transcriptional CDKs

Whereas the transcriptional functions of several CDKs and cyclins are clearly established, others are still under investigation [14]. CDK7 associates with Cyclin H to form a complex termed CAK, the CDK-Activating Kinase, which is involved in activation of several CDK/Cyclins through phosphorylation of the CDK activation loop (or T-loop). In addition, CDK7 plays an essential role in transcription by contributing to activating phosphorylation of RNA polymerase II. Aside from CDK7, several other CDKs have been found to be implicated in transcriptional processes [50,71]. 

CDK3 associates with Cyclin C and plays a role in regulation of Rb-dependent G0/G1 transition [39]. CDK3 also phosphorylates activating transcription factor 1 (ATF1), thereby enhancing its transactivation and transcriptional activities [40].

CDK8 is a component of Mediator, an evolutionary conserved multiprotein complex that regulates RNA polymerase II-dependent transcription. Besides its role in regulation of gene transcription, it has been implicated as a regulator of multiple steps in cell cycle progression [51].

CDK9 associates with cyclin T1 and plays a role in transcription regulation through regulation of RNA polymerase II [72]. CDK9/Cyclin T1 was in fact identified as the PTEFb transcription factor and further found to bind HIV TAT and promote its transactivation from the integrated provirus through activation of RNA polII. As such, CDK9 has emerged as a novel target for anti-retroviral strategies. 

CDK10 was initially identified in regulation of G2, but was more recently found to associate with Cyclin M and behave as a transcriptional activator through phosphorylation of Ets transcription factor [57,58].

#### 1.1.3. CDK5—Neuronal and Non Neuronal Functions

CDK5 is an unconventional member of the CDK family which is ubiquitously expressed, but mainly active in post-mitotic neurons, where it is activated by neuron-specific activators p35/p25 and p39 rather than by cyclins [73,74]. Initially identified through structural homology with CDK2 [75,76], and independently isolated as a proline-directed histone H1 kinase from bovine brain, CDK5/p35 was found to phosphorylate histone H1 and retinoblastoma protein (pRb) [77,78,79]. However, this kinase is not involved in coordination of cell cycle progression, and instead exerts its functions mainly in the central nervous system, where it promotes neurite extension, neuronal migration, synapse formation during brain development, synaptic plasticity and synaptic activities in mature neurons, axonal guidance, neuronal development and differentiation, and further participates in regulation of autophagy [43,44,45,80,81,82,83,84]. Moreover several extraneuronal functions of CDK5 have been reported including apoptosis in nonneuronal model systems and insulin secretion in pancreatic cells [46,85,86].

CDK5 activation occurs following Ca^2+^-stimulated calpain-dependent proteolytic cleavage of p35 or p39 proteins to yield a p25 protein responsible for complete activation of CDK5 [87,88,89] (Figure 2b). p25 does not share any primary sequence similarity with cyclins but adopt a cyclin-box fold and binds CDK5 at the interface through which CDKs interact with cyclins, and in a similar fashion to Cyclin A binding to CDK2 [90,91].

*In vitro*, CDK5/p35 and CDK5/p25 have been shown to exhibit very similar kinase activity towards histone H1 and tau [92]. However, a 10 kDa *N*-terminal myristoylated sequence in p35 anchors the CDK5/p35 complex to the cell membrane, and release into the cytoplasm requires cleavage of p35 to p25 [89,93]. Moreover, activation of CDK5 by p25 *in cellulo*, following stress or neurotoxic signals is greater than the activation of CDK5 by p35 [94]. 

CDK5 exerts a double protective function in neurons by shuttling between the nucleus, where it localizes through interaction with p27 and suppresses cell cycle progression by disrupting the DP1-E2F1 dimer and its DNA binding ability, and the cytoplasm, where it is involved in inactivation of signaling pathways leading to cell death [95,96] (Figure 2B). 

#### 1.1.4. Other Non Cell Cycle CDKs

Research over the last decade has revealed that members of the CDK/Cyclin family are involved in a much broader spectrum of biological functions than initially suspected. These functions range from processes associated with genetic material, such as DNA synthesis and replication, epigenetic regulation, DNA damage response and repair, transcriptional regulation, and functions associated with development and differentiation, including neuronal differentiation, synaptic trafficking and remodelling, glycogen synthesis and lipogenesis, angiogenesis, hematopoiesis, ciliogenesis and spermatogenesis ([14] and references therein). Established functions as well as ongoing functional characterization of CDK functions have been very well described eslewhere and will therefore not be discussed further here [13,14,15,16]. 

#### 1.1.5. Functional Redundancy of CDK/Cyclins

Despite a high level of coordination and specificity underlying formation and activation of CDK/cyclin complexes, functional redundancy has been observed between CDKs and Cyclin partners. Indeed, knockout studies in mice reveal that the only indispensable CDK is CDK1, capable of compensating for the lack of interphase CDKs and recapitulating all cell cycle transitions and driving progression through the cycle in mammalian cells through sequential association with different cyclins, The absence of other cell cycle CDKs or cyclins, except for cyclins B1 and A2, is completely bypassed and compensated in mammalian cells through formation of “illegitimate” complexes which do not normally occur in physiological conditions [15,36,97,98,99]. Studies in mouse models have revealed that although each phase of the cell cycle is driven by a specific CDK, all interphase CDKs including CDK2, CDK4 and CDK6 are dispensable for cell cycle progression in most cell types [36,97]. These CDKs are however essential for development and differentiation of highly specialized cell types. 

### 1.2. Structure and Regulation of CDK/Cyclins

In mammalian cells, monomeric CDKs are inactive and require association with a regulatory cyclin subunit to acquire a stable and active conformation [100,101]. The spatio-temporal pattern of expression and degradation of cyclins, named after their cyclical pattern of synthesis and degradation, together with their structural and molecular features, dictates their ability and availibity to interact with a CDK, and thereby determines the orderly formation of different CDK/Cyclin complexes throughout the cell cycle or in specific functional pathways. Cyclins further regulate the subcellular localization of CDKs, thanks to NES or NLS sequences which enable their nucleo-cytoplasmic shuttling. Moreover cyclins contribute to substrate recruitment through specific docking sites [102]. Activation of CDK/Cyclin complexes is then subject to several levels of control, including reversible activating and inhibitory phosphorylations as well as interactions with structural inhibitors [11,12,103]. Premature CDK activation is first controlled through phosphorylation of residues that line the nucleotide-binding pocket, which are believed to prevent ATP binding, specifically Thr14 and Tyr15 for CDK1 and CDK2, by Wee1 and Myt1 kinases [103,104,105]. These inhibitory phosphorylations are removed through activation of Cdc25 phosphatases [106], which act in concert with phosphorylation by the CDK-activating kinase CAK (on Thr160 for CDK2) [11,12,71]. 

Structural inhibitors of CDKs (CKIs) bind CDKs or CDK/Cyclin complexes thereby sequestering them and constraining their conformations [107,108,109,110]. INK4 inhibitors, p16, p15, p18 and p19, are composed by multiple ankyrin repeats which bind either monomeric CDK4/6 or CDK4/6-cyclin D complexes [107]. CIP/KIP inhibitors, p21^Cip1^, p27^Kip1^, p57^Kip2^, comprise characteristic motifs bind and inhibit CDK2/Cyclin A/E, yet have been reported to promote assembly and nuclear import of CDK4/cyclin D [109,110].

**Figure 3:**
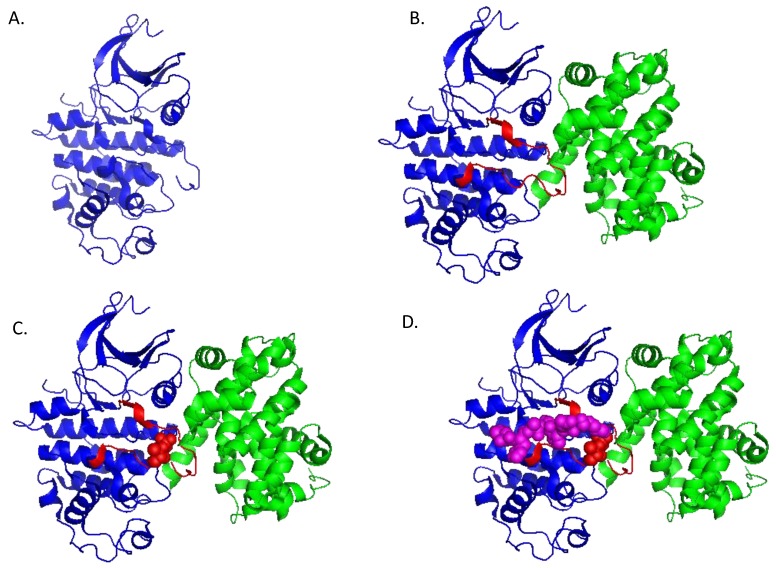
Structures of monomeric CDK2 and CDK2/CyclinA. (**A**) Structural representation of CDK2; (**B**) Structural representation of CDK2/Cyclin A complex (PDB: 1QMZ); (**C**) Structural representation of phospho Thr160-CDK2/Cyclin A; (**D**) Structural representation of phospho Thr160-CDK2/Cyclin A bound to a peptide substrate.

The general structure of CDKs is conserved throughout this family of kinases, with a characteristic of bilobe fold harbouring a conserved ATP-binding pocket within the *N*-terminal lobe, close to the catalytic cleft located between the two lobes (Figure 3A). Major structural features do not vary significantly, although the nature of surface residues may vary significantly, and plays an important role in defining substrate and partner specificity [111]. Likewise, cyclins are characterized by a compact alpha helix rich cyclin-fold, with variations in the length and relative position of the helices as well as in the nature of surface residues involved in interactions with CDK, substrates, or specific partners [112,113] (Figure 3B).

The primary interaction between CDKs and Cyclins involves a conserved structural element, known as the PSTAIRE helix in CDK2 (or C helix) and alpha helix 5 of the cyclin. The specificity and affinity between two partners further implicates molecular determinants in the *C*-terminal lobe of the CDK and other alpha helices (Nter and alpha3) [101,114]. Cyclin binding to a CDK induces major conformational changes in the kinase subunit, which were first highlighted when the crystal structure of CDK2/Cyclin A was solved, and further evidenced through mechanistic and kinetic studies of CDK2/Cyclin A assembly [101,115]. Cyclin binding induces reorientation of the ATP-binding pocket so as to align it with the catalytic cleft, thereby favouring coupling of ATP hydrolysis with phosphate transfer onto the substrate. Moreover, cyclin A induces a conformational switch of the activation segment (or T-loop) of CDK2, thereby rendering the substrate binding cleft more accessible and exposing this loop for phosphorylation by the CDK-Activating Kinase CAK (Figure 3B). Phosphorylation of CDK2 on Thr160 further stabilizes the T-loop, yielding a fully accessible substrate-binding site, and thereby ameliorating catalysis of the phosphotransfer reaction [116,117,118] (Figure 3C,D).

## 2. Cyclin-Dependent Kinases in Cancer

In physiological conditions, activation of CDK/Cyclin kinases is tightly controlled both spatially and temporally. However, CDK/Cyclins are dysregulated in several human cancers, which wreaks havoc in the coordinated cycle of cell growth and proliferation and contributes to the uncontrolled proliferation characteristic of cancer cells [15,119,120,121]. In fact, together with mutations in proto-oncogenes, mutations leading to hyperactivation of CDK activity have been reportedly found in human cancer genomes, and confer selective growth advantage to cells, whilst mutations that inactivate checkpoint regulators, tumour suppressor genes or CKIs result in loss of cell cycle inhibition [15,122,123,124,125,126,127].

**Figure 4:**
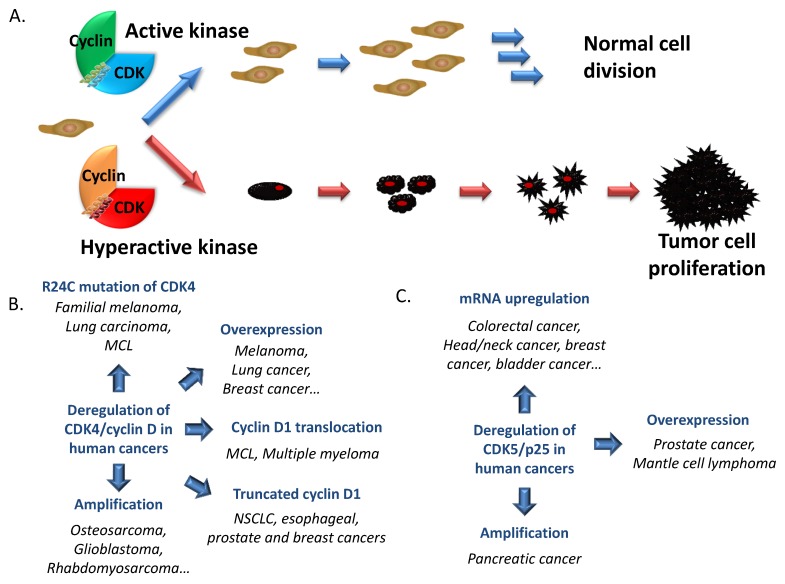
CDK/Cyclins and Cancer. (**A**) Schematic representation of normal cell growth and division regulated by cyclin-dependent kinases. Hyperactivation of these kinases contributes to development of cancer cell proliferation; (**B**) CDK4/cyclin D in cancers: Among all the CDK/cyclins, the complex CDK4/cyclin D is the one which presents most aberrations in cancers. Hyperactive CDK4/cyclin D is found in several human cancers associated with the R24C mutation of CDK4 which prevents the fixation of the endogenous inhibitor p16^INK4A^, mutation of p16^INK4a^ itself or CDK4 or cyclin D amplification; (**C**) CDK5/p25 in cancers.

CDK/Cyclin hyperactivation may result from one of several causes, including gene amplification and protein overexpression of either the CDK or cyclin subunit, alternative splicing and expression of truncated cyclin variants, untimely expression and mislocalization, or constitutive activation of CDK/Cyclins by preventing their inactivation through binding to INK or KIP/CIP inhibitors [15,121,128,129,130,131,132,133,134]. A representative panel of mutations which occur in CDKs and Cyclins may be found in the catalogue of Cosmic Mutations in Cancer (COSMIC database—http://www.sanger.ac.uk/genetics/CGP/cosmic/) [135], which integrates all mutations identified through sequencing of human cancer tissue samples. Figure 4A schematizes how alterations in CDK/Cyclin function contribute to establishment of cancer phenotypes. Table 2 summarizes the different mutations and dysregulations in CDK and Cyclin expression associated with human cancers.

### 2.1. Cell Cycle CDKs

The mitotic kinase CDK1/Cyclin B being essential for viability, and capable of recapitulating the functions of the other cyclin-dependent kinases in regulating cell cycle transitions in mammalian cells is not surprisingly one of the least mutated kinases in human cancers. One single missense mutation of CDK1 has been reported in the COSMIC database in ovary carcinoma at amino acid position 73 (aspartic acid to histidine) [135]. CDK1 overexpression has been documented in lymphoma, advanced melanoma and lung cancer, and loss of cytoplasmic CDK1 predicts poor survival and confers chemotherapeutic resistance in the latter [136,137,138]. Cyclin B1 overexpression and/or mislocalization has been described in several primary cancers including breast, colon, gastric, prostate, thyroid carcinoma and non small-cell lung cancer (NSCLC) [139,140,141,142,143].

CDK2 overexpression has been reported in laryngeal squamous cell cancer, advanced melanoma and breast cancer [137,144,145,146]. Moreover, 33 simple coding mutations have been reported in the COSMIC database for CDK2 in a wide variety of cancer tissues, most of which are missense mutations in the Nterminal lobe (amino acid 2, 13, 20, 34, 45, 68, 84), one silent mutation at amino acid 45 and one deletion frameshift at amino acid 79 [135]. But CDK2 hyperactivation in human cancers is most often associated with amplification and/or overexpression of its partner cyclins A and E in a wide variety of human cancers, but in particular in breast cancer, ovarian and endometrial carcinomas, lung and thyroid carcinoma, melanoma and osteosarcoma [129,131,143,147,148,149,150,151,152,153,154,155,156,157,158]. 

Cyclin A overexpression correlates with carcinogenesis and metastasis in colorectal cancers, and constitutes an independent prognostic factor in patients with colorectal adenocarcinoma [147,159,160]; Cyclin E amplification and overexpression has also been documented in these cancers [147,161]. Moreover, integration of hepatitis B virus into cyclin A gene results in expression of a truncated form which has been reported in hepatocellular carcinoma [151,162]. Recent genomic analyses have identified cyclin E1 gene amplification as a candidate oncogenic driver in high-grade serous ovarian carcinoma [154]. Cyclin E1 dysregulation has further been reported to drive malignant transformation in fallopian tube secretory cells that are the site of origin of high-grade serous ovarian carcinomas [155]. 

**Table 2:** CDK and cyclin aberrations associated with cancer.

Target	Deregulation	Cancer	Reference
**CDK1**	Overexpression	B lymphoma, advanced melanoma	[136,137]
	1 simple coding mutation/missense mutation (D73H)	ovary carcinoma	[135]
**CDK2**	Overexpression	Laryngeal squamous cell cancer, advanced melanoma, breast cancer	[137,144,145,147]
	33 simple coding mutation/25 missense mutations/7 synonymous mutations/1 frameshift mutation	wide variety of cancer tissues	[135]
**CDK3**	overexpression	glioblastoma	[40]
	1 simple coding mutation/missense mutations (S106N)	glioma	[135]
**CDK4**	Amplification	refractory rhabdomyosarcoma, osteosarcoma, glioblastoma	[163,164,165]
	Overexpression	melanoma	[166]
	Overexpression	lung cancer	[167]
	Amplification/Overexpression	osteosarcoma, sporadic breast carcinoma, uterine cervix cancer	[164,168,169]
	R24C mutation	Familial melanoma	[170,171,172,173,174,175,176,177]
	R24C mutation	lung carcinoma	[178]
	R24C mutation	mantle cell lymphoma	[179]
	38 simple coding mutation/25 missense mutations/12 synonymous mutations	wide variety of cancer tissues	[135]
**CDK5**	Amplification/Overexpression	Pancreatic cancer	[180]
	Overexpression	Breast cancer	[181]
	Decreased methylation of promoter leading to overexpression	mantle cell lymphoma	[182]
	Single nucleotide polymorphisms (SNPs) in the promoter region	increased risk of lung cancer	[183]
	Overexpression	Prostate cancer	[184]
	mRNA upregulation	colorectal, head/neck, breast, lung, ovarian, lymphoma, prostatic, sarcoma, myeloma and bladder cancers	[185]
	24 simple coding mutation/15 missense mutations/7 synonymous mutations/1 deletion frameshift	wide variety of cancer tissues	[135]
**CDK6**	translocation	splenic marginal zone lymphoma	[186]
	amplification	squamous cell carcinoma, glioma and lymphoma	[187,188]
	D32Y mutation	neuroblastoma	[189]
	sumoylation	glioblastoma	[190]
	overexpression	medulloblastoma	[191]
	33 simple coding mutation/1 nonsensense substitution/18 missense mutations/11 synonymous mutations/1 complex mutation	wide variety of cancer tissues	[135]
**CDK7**	24 simple coding mutation/1 nonsensense substitution/19 missense mutations/3 synonymous mutations	wide variety of cancer tissues	[135]
**CDK8**	overexpression	colon cancer	[192]
	amplification and overexpression	colorectal cancer	[52,193,194]
	gastric	gastric cancers	[195]
	upregulation upon loss of macroH2A histone variant	melanoma	[196]
	siRNA-mediated silencing inhibits proliferation	breast cancer	[197]
	tumor-suppressive function	endometrial cancer	[198]
	65 simple coding mutation/9 nonsensense substitution/42 missense mutations/12 synonymous mutations/2 inframe deletions	wide variety of cancer tissues	[135]
**CDK9**	highly expressed	chronic lymphocytic leukemia and multiple myeloma	[199]
	differential expression correlating with lymphoid differentiation/activation and malignant transformation	lymphoma	[200]
	expression correlates with differentiation grade	neuroblastoma and primary neuroectodermal tumours	[201]
	1 simple coding mutation/missense mutation (D323N)	lung adenocarcinoma	[135]
**CDK10**	downregulation	biliary tract cancer	[202]
	downregulation	hepatocellular carcinoma	[203]
	1 simple coding mutation/missense mutation (N157S)	ovary carcinoma	[135]
**CDK11**	Gene deletion/translocation	neuroblastoma	[204]
	Loss of one allele of Cdc2L/reduced CDK11 expression	melanoma	[205]
	overexpression	osteosarcoma	[206]
	essential for growth of liposarcoma cells	liposarcoma	[207]
**CDK11A**	43 simple coding mutation/2 nonsensense substitution/30 missense mutations/8 synonymous mutations/2 inframe deletions	wide variety of cancer tissues	[135]
**CDK11B**	38 simple coding mutation/2 nonsensense substitution/21 missense mutations/12 synonymous mutations/2 inframe insertions/2 deletion frameshifts	wide variety of cancer tissues	[135]
**CDK12**	189 simple coding mutation/17 nonsensense substitution/123 missense mutations/30 synonymous mutations/5 frameshift insertions/2 inframe deletions/11 deletion frameshifts/2 complex	wide variety of cancer tissues	[135]
**CDK13**	124 simple coding mutation/4 nonsensense substitution/96 missense mutations/22 synonymous mutations/1 inframe deletions/5 deletion frameshifts/1 complex	wide variety of cancer tissues	[135]
**CDK14**	overexpression associated with increased cell migratory properties	hepatocellular carcinoma	[208]
	overexpression associated with enhanced of chemoresistance	oesophageal squamous cell carcinoma	[209]
	92 simple coding mutation/3 nonsensense substitution/62 missense mutations/20 synonymous mutations/1 inframe deletions/1 deletion frameshift	wide variety of cancer tissues	[135]
**CDK15**	68 simple coding mutation/4 nonsensense substitution/42 missense mutations/14 synonymous mutations/3 deletion frameshifts	wide variety of cancer tissues	[135]
**CDK16**	35 simple coding mutation/1 nonsensense substituation/29 missense mutations/4 synonymous mutations	wide variety of cancer tissues	[135]
**CDK17**	76 simple coding mutation/7 nonsensense substituation/47 missense mutations/13 synonymous mutations/1 deletion frameshift/1 complex	wide variety of cancer tissues	[135]
**CDK18**	48 simple coding mutation/1 nonsensense substituation/28 missense mutations/19 synonymous mutations/1 deletion frameshift	wide variety of cancer tissues	[135]
**CDK19**	65 simple coding mutation/1 nonsensense substituation/45 missense mutations/16 synonymous mutations/1 deletion frameshift	wide variety of cancer tissues	[135]
**CDK20**	1 simple coding mutation/missense mutation (A165V)	malignant melanoma	[135]
**Cyclin A**	overexpression	esophageal squamous cell carcinoma, acute myeloid leukemia, soft tissue sarcoma, hepatocellular carcinoma, thyroid carcinoma, endometrial adenocarcinoma	[131,143,148,150,151,152]
	overexpression	colorectal cancer	[147,159,160]
	amplification	breast cancer	[129]
	truncated form due to integration of hepatitis B virus DNA	hepatocellular carcinoma	[151,162]
**Cyclin B**	Overexpression	breast cancers, esophageal squamous cell carcinoma, NSCLC, thyroid carcinoma	[139,140,141,142,143]
	overexpression/nuclear localization	breast cancer	[132]
**Cyclin D**	Overexpression	Follicular mantle cell lymphoma, lung cancer, breast cancer, head and neck, esophageal cancer	[133]
	Overexpression	Colorectal adenocarcinomas	[210]
	Overexpression	lung cancer	[167]
	Overexpression	pancreatic cancer	[211]
	Overexpression	endometrial carcinoma	[212]
	Amplification/overexpression	head and neck carcinoma	[213,214,215]
	IGH translocation and overexpression	multiple myeloma	[216,217]
	IGH translocation and overexpression	mantle cell lymphoma	[218,219]
	Mutation that disrupts phosphorylation-dependent nuclear export	Esophageal cancer	[220]
	Truncated form (cyclin D1b) (A870G polymorphism)/Nuclear Accumulation	NSCLC	[221,222,223]
	Truncated form (cyclin D1b) (A870G polymorphism)/Nuclear Accumulation	esophageal and prostate cancer	[224,225]
	Truncated form (cyclin D1b) (A870G polymorphism)/Nuclear Accumulation	prostate cancer	[226]
	Truncated form (cyclin D1b) (A870G polymorphism)/Nuclear Accumulation	breast cancer	[227]
	Truncated form (cyclin D1b) and its co-expression with cyclin D1a	breast cancer	[228]
	cyclin D1a isoforms with truncated 3' UTRs, not alternatively spliced cyclin D1b mRNA isoforms/alterations of *CCND1* 3' UTR structure	mantle cell lymphoma	[229]
**Cyclin E**	amplification	ovarian cancers	[154,155]
	Overexpression	acute and chronic leukemias, Hodgkin’s and non-Hodgkin’s lymphomas	[230,231]
	Overexpression	osteosarcoma, NSCLC, pancreatic cancer	[156,157,158]
	Overexpression/amplification	colorectal cancer	[147,161]
	Overexpression/High nuclear expression	early development of breast cancers	[153]
	Overexpression of small isoforms	breast cancers	[145,232,233,234]
	Low mol weight (LMW) isoform (truncated)	breast cancer, melanoma, ovarian carcinoma tumors	[130,235,236,237]

High levels of Cyclin E in the nucleus have been reported to occur at an early stage in development of breast cancer [153], and low molecular weight forms of cyclin E are thought to be responsible for hyperactivation of CDK2 in breast cancer, melanoma and ovarian carcinoma [130,232,233,235,236,237,238]. Cyclin E overexpression has further been described in leukemias and lymphomas, osteosarcoma, pancreatic cancer and NSCLC [156,157,158,230,231].

Deregulation of CDK4 and CDK6 kinase activities associated with D-cyclins resulting in Rb hyperphosphorylation is associated with a loss of control between mitogenic stimuli and cell cycle regulation, which leads to uncontrolled cell proliferation. CDK4 hyperactivity has been well documented in a wide variety of cancers, and in particular in melanoma, lung cancer and lymphoma [173,174,175,178,179] (Figure 4A). In fact genetic alterations in components of the pRb/CDK4/cyclin D/p16^INK4a^ pathway are amongst the most frequently occurring anomalies reported, found in more than half of all human tumours [238]. Genetic inactivation of p16^INK4a^ following deletion or mutation of the gene encoding this structural inhibitor of CDK4/Cyclin D is one of the most frequent tumour suppressor mutations in human cancers and results in defective inhibition of CDK4. Likewise, the CDK4 R24C point mutation confers selective growth advantage by causing loss of CDK4 binding to p16^INK4a^, and consequently constitutive activation of CDK4 in familial melanoma and in a subset of lung cancers and lymphomas [173,174,175,178,179,238]. In fact, the importance of CDK4 protein in human cancers was first highlighted upon identification of a germ line mutation (R24C) that predisposed to melanomas by making it refractory to inhibition by p16^INK4a^. Surprisingly, knockin of the CDK4 R24C mutation in mouse models revealed a very low incidence of spontaneous melanomas in CDK4^R24C/R24C^ mice. However, an increased incidence of spontaneous melanomas was observed in mice expressing the HRAS (G12V) oncogene in melanocytes in the R24C background [175]. Gene amplification and overexpression of CDK4 have been reported in sporadic melanoma and sporadic breast carcinoma, in refractory rhabdomyosarcoma, osteosarcoma, liposarcoma, glioblastoma and neuroblastoma [163,164,165,168,189,238,239,240,241,242]. The COSMIC database reports on 38 simple coding mutations in CDK4, with 25 missense mutations within its *N*-terminal lobe (residues 2, 9, 10, 23, 24, 31, 35, 43) and 12 synonymous mutations encoding silent substitutions at positions 2 and 18 [135]. CDK4 hyperactivity is further often associated with Cyclin D overexpression, genetic amplification, polymorphism, translocation or alternative splicing [134,238]. High levels of cyclin D1 are frequently encountered in breast cancers either through genetic amplification or overexpression [243]. Genetic aberrations of cyclin D1 (*CCND1*), including overexpression, have been reported in neuroblastoma [240]. Cyclin D1 overexpression alone is not sufficient to drive oncogenic transformation, but its nuclear accumulation is clearly associated with neoplastic development [133]. Somatic mutations of cyclin D1 are rare, however a specific polymorphism (A870G) yields an alternatively spliced transcript, cyclin D1b, which lacks a specific phosphorylation site required for nuclear export and therefore accumulates in the nucleus, and is associated with increased cancer risk and outcome [224]. The p16^INK4a^-cyclin D-CDK4/6-retinoblastoma protein pathway (CDK4 pathway) is dysregulated in 90% of melanomas. In both human and mouse models of melanoma, activation of the CDK4 pathway potently cooperates with mutant BRAF or NRAS in transformation of melanocytes and RAS/RAF/MEK/ERK pathway is dysregulated in 65% to 90% of metastatic melanoma, further enhancing CDK4 pathway signaling through increasing cyclin D1 expression [175,244]. The risk of developing melanomas is also increased by CDK4/cyclin D hyperactivation associated with amplification of cyclin D (18% melanomas) or loss of p16^INK4a^ inhibitor of CDK4/cyclin D (deletion of *CDK2NA* in 50%–60% metastatic tumours) [176]. This kinase therefore constitutes an attractive pharmacological target for melanoma therapeutics [177]. Dysregulation of the p16^INK4a^/CDK4/CyclinD pathway is equally frequent in lung cancers. Cyclin D1 gene is amplified in non-small cell lung cancer (NSCLC) and cyclin D1 protein is frequently overexpressed in tumours and pre-invasive bronchial lesions [221]. CDK4/Cyclin D1 overexpression is an indicator of prognosis in human primary lung carcinoma [167]. Furthermore, the discovery of a synthetic lethal interaction between K-Ras oncogenes and CDK4 in a mouse tumour model of NSCLC revealed that KRAS-driven NSCLC is particularly dependent on CDK4 [178]. Furthermore, targeting CDK4 alleles in advanced tumours of this KRAS-mutant model induced senescence and prevented tumour progression, thereby highlighting the pharmacological importance of CDK4 for therapeutic strategies [178].

CDK6 gene amplification and overexpression have been described in lymphomas, leukemias, squamous cell carcinoma, gliomas and medulloblastoma [186,187,191]. This overexpression can lead to translocation of CDK6 in some leukemias, and may link the TP53 and RB1 tumor suppressor pathways to medulloblastoma pathomechanisms [191]. The COSMIC database reports on 33 simple coding mutations in CDK6, with 1 nonsense substitution at position 157, 18 missense mutations (amino acids 18, 84, 87, 113, 118 and 139) and 11 synonymous mutations that encode silent substitutions at positions 65 and 148 [135].

### 2.2. CDK5

CDK5 hyperactivity is associated with the onset and development of neurodegenerative disorders, inducing neuronal cell death [47,82,245]. Several studies have shown that CDK5 is hyperactivated by p25 in Alzheimer’s disease, amyotrophic lateral sclerosis (ALS), and Parkinson’s disease [47,245,246,247,248,249,250]. Indeed increased activity of CDK5 contributes to Tau hyperphosphorylation and consequently to formation of intracellular neurofibrillary tangles observed in the brain of Alzheimer’s patients [245,246,248,250,251,252,253]. Moreover, CDK5 participates in the hyperphosphorylation of alpha-synuclein and parkin, thereby contributing to generation of Lewy bodies in Parkinson’s disease [254,255] and to Lewy bodylike inclusions, which ultimately contribute to neuronal loss in amyotrophic lateral sclerosis [247,256]. 

Aside from its contribution to neurodegenerative diseases, numerous studies indicate that CDK5 also constitutes a relevant target in oncology (reviewed in [85]) (Figure 4B). CDK5 hyperactivation would seemingly contribute to development of glioblastoma and neuroblastoma [257]. Moreover significant upregulation of CDK5 expression has been reported in colorectal, head/neck, breast, lung, ovarian, lymphoma, prostate, sarcoma, myeloma and bladder cancers, and there is well documented evidence that it plays a role in these cancers as well as lymphoma and multiple myeloma [180,181,182,183,184,185,258,259,260]. The level of CDK5 expression predicts the survival of relapsed multiple myeloma patients [257]. Patients with lung cancer expressing CDK5/p35 have a poorer prognosis than those that do not express [146]. CDK5 was found to play an important role in regulation of cell motility, migration and metastasis in prostate cancer cells [184]. Single nucleotide polymorphisms (SNPs) in the promoter region of the cdk5 gene have been linked to increased risk for lung cancer [183]. Decreased methylation of the cdk5 promoter region, which resulted in increased cdk5 expression, was also observed in mantle cell lymphoma [182]. CDK5 and its main activators, p35 and p39 are rarely expressed in pancreatic ducts, but found to be highly expressed due to gene amplification in 67% human pancreatic ductal adenocarcinoma, and was further reported to act in concert with K-Ras to promote malignant progression, migration and invasion of pancreatic cancer cells [180,260]. In breast cancer CDK5 participates in TGF-β1-induced epithelial-mesenchymal transition and TGF-β1 upregulates CDK5 and p35 expression [181]. The COSMIC database reports on 24 simple coding mutations in CDK5, most of which are missense substitutions (amino acids 16, 31, 48, 50, 73 and 101) one deletion frameshift at position 23 and seven synonymous mutations which encode two silent substitutions at positions 16 and 67 [135].

### 2.3. Transcriptional CDKs

Aberrations in transcriptional CDKs have also been reported in several human cancers, with direct consequences on the upregulation of target genes. 

CDK8 has been reported as an oncoprotein in colorectal and gastric cancers and further evidence points to its role in promoting cell proliferation in melanoma and breast cancer [52,261,262,263]. CDK8 kinase activity is required for β-catenin-driven transformation and expression of several β-catenin transcriptional targets. Colorectal cancers with CDK8 expression have distinct clinical, prognostic and molecular attributes and both CDK8 and β-catenin levels have been reported to correlate with carcinogenesis, tumor progression and increased colon cancer-specific mortality [193,194]. However suppression of CDK8 expression inhibits proliferation in colon cancer cells characterized by high levels of CDK8 and β-catenin hyperactivity [52]. Likewise, CDK8 was found to be overexpressed in gastric adenocarcinomas and CDK8 expression and the delocalization of β-catenin expression showed a significant positive correlation with carcinogenesis and tumor progression [195]. Upregulation of CDK8 expression was also described in melanoma following loss of the histone variant macroH2A [196]. A role for CDK8 in breast cancer has been suggested through siRNA silencing in breast cancer cell lines which leads to a significant decrease in proliferation [197]. CDK8 is further required for tumor growth and maintenance of tumor dedifferentiation *in vivo* and has been reported to play a role in control of cancer and stem cell function. CDK8 expression correlates with embryonic stem cell pluripotency and loss of CDK8 causes embryonic stem cells to differentiate through regulation of Myc target gene expression. Likewise, increased expression of a CDK8-regulated, embryonic stem cell MYC target gene signature was associated with loss of differentiation and poor outcome in primary human colon cancers [192]. 

CDK9-signaling pathways are involved in development of tumorigenesis and abnormal CDK9/cyclin T1 activity has been described in several human malignancies [264]. Deregulation of CDK9/cyclin T1 activity is essentially associated with its overexpression in several B and T-cell lymphomas, as well as in neuroblastoma, primary neuroectodermal tumor, rhabdomyosarcoma and prostate cancer [200,201,264,265,266].

Overexpression of CDK9 and cyclin T1 has been reported in B and T cell precursor-derived lymphomas, anaplastic large T cell lymphoma, and follicular lymphomas, while strong nuclear staining of these proteins has been described in classical Hodgkin’s lymphoma [200]. Abnormal mRNA levels of CDK9 and cyclin T1 have been found in Burkitt’s lymphoma, diffuse large B cell lymphoma with germinal center phenotype, classical Hodgkin’s lymphoma-derived cell lines, and follicular lymphoma [200]. CDK9 is further deregulated in myeloid leukemia [199]. Moreover, CDK9 expression levels have been found to correlate with the differentiation grade of neuroblastoma and primary neuroectodermal tumours [201].

CDK3 protein expression levels have been reported to be higher in human cancer cell lines and human glioblastoma tissue compared with normal brain tissue, and its function in cell proliferation and transformation appears to be linked to signalling of the transcriptional activator ATF1 [40].

Aside from its recent implication in STAR syndrome [58], CDK10 has been reported as a tumour suppressor and an important determinant of resistance to endocrine therapy for breast cancer [59]. CDK10 downregulation has been reported in biliary tract tumors and cell lines, whereas its overexpression causes malignant cells to become resistant to chemotherapy [202]. Similarly, CDK10 mRNA and protein levels are decreased in hepatocarcinoma samples compared to adjacent nontumorous liver tissues [203].

Although the function of CDK11 remains unclear in tumour development, alterations of one of the major isoforms CDK11^p110^ have been reported in several tumour cell lines [204,206,207]. Deletion or translocation of CDK11 gene have been reported in neuroblastoma [204]. CDK11 is highly expressed in osteosarcoma and would be essential for osteosarcoma cell growth and survival [206]. CDK11 has been reported to be essential for growth and proliferation of liposarcoma cells, since its knockdown decreased cell proliferation and promoted apoptosis [207].

## 3. Targeting Cyclin-Dependent Kinases—Strategies and Inhibitors

Human cancers are characterized by altered cell cycle regulation. Dysfunctions in the mechanisms that coordinate cell cycle progression are intimately related to the characteristic features of cancer cells as defined by Hanahan and Weinberg [119]. In particular, self-sufficiency to growth factors and insensitivity to anti-growth factors are typically associated to uncoupling of CDK/Cyclin functions and checkpoint response with the tight coordination of cell cycle progression. Indeed CDK/Cyclin kinase hyperactivity is frequently observed in human cancers, and the molecular causes of these dysregulations are well characterized and in most instances associated with tumour progression and poor prognosis in patients. As such, cyclin-dependent kinases constitute attractive pharmacological targets for the development of anticancer drugs and targeting CDKs has been pursued as a strategy for therapeutic intervention since the late 1980s [206,207,267,268,269,270,271,272,273,274,275,276]. 

Whilst traditional strategies have aimed at targeting cancer cells by tampering with DNA integrity or replication through administration of alkylating agents, anti-metabolites, topoisomerase inhibitors, or inhibitors that target mitotic spindle assembly/disassembly, more recent targeted strategies have focused on development of inhibitors of the kinases which are essential and directly responsible for cellular aberrations [276]. Targeting CDK1/Cyclin B, its essential mitotic functions and unique ability to compensate for all other cell cycle cyclin-dependent kinases constitutes an attractive means of inhibiting cell proliferation. Strategies aimed at targeting CDK1 and cyclin B have been proposed and effectively shown to block growth of cancer cells and tumours [277,278,279,280,281,282,283]. Likewise, targeting the functions of CDK2 in DNA replication and S phase progression, provides a window of intervention for cancer therapeutics. CDK4/Cyclin D kinase constitutes an established pharmacological target in several human cancers, in particular in melanoma and in KRAS-mutant NSLCL lung cancers [177,178].

**Figure 5:**
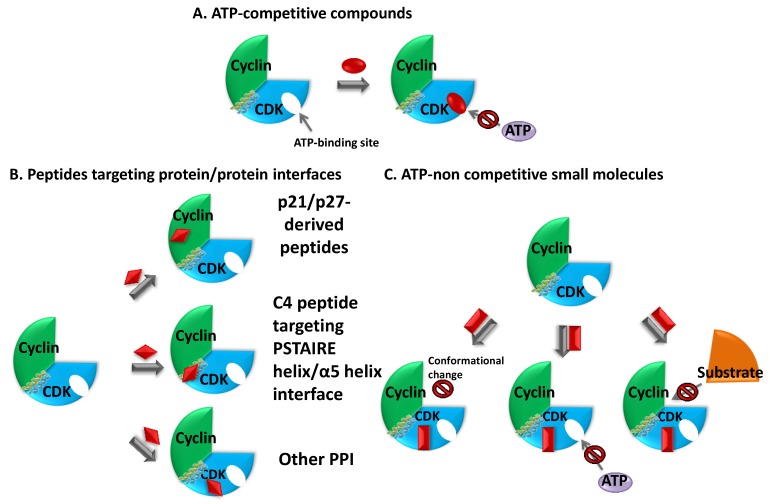
Strategies for targeting Cyclin-dependent Kinases. (**A**) ATP-competitive inhibitors bind the ATP pocket and compete with ATP binding; (**B**) Protein-protein interface inhibitors target essential and specific protein/protein interactions either between CDKs and Cyclins, or between CDK/Cyclins and p21/p27/p107 proteins-targeting the cyclin-binding groove. The example shown here represents an inhibitor targeting the primary interface between the CDK and the cyclin [the “PSTAIRE” helix of the CDK and alpha 5 helix of the cyclin]. (**C**) Allosteric inhibitors preventing ATP binding target sites which are remote from the ATP-pocket, so as to stabilize enzymatically inactive conformations or interfere with conformational transitions associated with kinase activation, compete with substrate or ATP binding.

Efforts aimed at targeting cyclin-dependent kinase hyperactivity in human cancers began through purification of compounds from natural sources. These first generation ATP-competitive compounds served as templates for structure-guided, rational design of second generation drugs that bind the ATP-binding pocket of CDKs. Moreover this class of drugs was implemented by compounds identified in activity-based screens of chemical libraries. Over the more recent years, alternative strategies have been sought to develop compounds targeting pockets and patches which are distinct from the ATP pocket, which are thought to offer greater promises of selectivity and therefore circumvent some of the undesired side-effects of ATP-competitive inhibitors [284,285,286,287,288,289]. Today, the list of CDK-Cyclin inhibitors comprises natural substances, ATP-competitive and non-competitive synthetic compounds with different chemical structures, as well as peptides and peptimimetics. They can be classified according to their specificity, as *pan*-specific or selective for one single CDK. They can further be described according to their mechanism of action, as ATP-competitive or non-competitive inhibitors—amongst the latter, substrate-competitive inhibitors and inhibitors of protein/protein interactions between CDKs and Cyclins, or between CDK/Cyclins and regulatory partners, and more recently allosteric inhibitors.These different classes of compounds will be described below in further detail. Figure 5 schematizes the different strategies which have been developed to target CDK/Cyclins and Table 3 lists some of the major inhibitors.

**Table 3:** Major CDK inhibitors.

Inhibitor	Type/Nature/Class	Target	References
**ATP-competitive compounds**			
Butyrolactone I	ATP-competitive/natural product	CDK1 > CDK2	[290,291,292,293]
Staurosporine	ATP-competitive/alkaloid	CDK1, CDK2, CDK4	[294,295]
7-hydroxystaurosporine/UCN01	ATP-competitive/alkaloid	CDK2, pRb, Chk1	[270,296,297]
Flavopiridol/Alvocidib	ATP-competitive/flavonoid	CDK2, CDK4, CDK6, CDK9	[270,298,299,300,301,302,303,304]
P276-00	ATP-competitive/flavone	CDK1, CDK4, CDK9	[305,306]
Hymenialdisine	ATP-competitive/natural product	CDK5, GSK3beta, CDK2, CDK1, Chk1	[307]
Fascaplysine	ATP-competitive/natural product	CDK4	[308]
Meriolins	ATP-competitive/aminopyrimidine indole	CDK1, CDK4, CDK9	[309]
Roscovitine/CYC202/Seliciclib/CYC065	ATP-competitive/trisubstituted purine	CDK5, CDK2, CDK1, CDK7, CDK9	[310,311,312,313,314,315]
NU2058 & NU6027	ATP-competitive/purine/pyrimidine	CDK1, CDK2	[316,317]
Purvalanol-A	ATP-competitive/purine	CDK1,2, 5	[318,319]
NU6140	ATP-competitive/purine		[320]
Olomoucine	ATP-competitive/purine	CDK1, CDK2, CDK5	[321,322]
Indirubin-5	ATP-competitive/indolinone	CDK1 > CDK2 > CDK5	[323,324]
SU9516	ATP-competitive/3-substituted indolinone	CDK2, CDK4	[325,326]
Paullones	ATP-competitive/paullone	CDKs	[323,324,327]
R547/Ro-4584820	ATP-competitive/Diaminopyrimidine	CDK1, CDK2, CDK4	[328,329,330]
Dinaciclib (SCH 727965)	ATP-competitive/pyrimidine	CDK9, CDK1, CDK2, CDK5	[331,332,333,334]
CDKI-73	ATP-competitive/pyrimidine	CDK9	[335]
PD-0183812	ATP-competitive/pyridine	CDK4, CDK6	[336]
PD-0322991/Palbociclib	ATP-competitive/pyrido-pyrimidine	CDK4, CDK6	[337,338,339,340,341,342]
LEE011/LY2835219	Small molecule	CDK1, CDK2, CDK4	[343,344,345]
SNS-032/BMS-387032	ATP-competitive/thiazole	CDK2, CDK7, CDK9	[346,347]
RO-3306	ATP-competitive/thiazolinone	CDK1 > CDK2	[348]
AT7519	ATP-competitive/pyrazole	CDK2, CDK9, CDK5, CDK4	[349,350,351,352]
**Peptides Targeting PPI**			
Spa310 and derivative from p130/pRb spacer domain	Peptide Competing with Substrate	CDK2/Cyclin A	[353,354]
CIP Peptide derived from p53, targeting CDK2/p53	Peptide Competing with Substrate	CDK2/Cyclin A	[355]
C4 interface peptide derived from Cyclin A	Peptide Targeting CDK/Cyclin PPI	CDK2/CyclinA	[356]
NBI1 hexapeptide targeting Cyclin A surface pocket	Peptide Targeting CDK/Cyclin PPI	CDK2/Cyclin A	[357]
Interface Peptides derived from p35: CIP and p5	Peptide Targeting CDK/Cyclin PPI	CDK5/p35	[358,359,360,361,362,363]
RXL peptides	Peptide Targeting Cyclin-binding Groove	CDK2/CyclinA	[364]
C-terminal hexapeptide PRGPRP	Peptides Targeting CDK4	CDK4/Cyclin D	[365]
Small peptides derived from E2F1	Peptide Targeting Cyclin-binding Groove	CDK2	[366]
Peptides derived from p21	Peptide Targeting Cyclin-binding Groove	CDK2, CDK4	[367,368,369,370,371,372]
Peptides derived from p27	Peptide Targeting Cyclin-binding Groove	CDK2, CDK4	[373]
Cyc103/cyclic peptide derived from p27	Peptide Targeting Cyclin-binding Groove	CDK2	[374]
Constrained peptidomimetic of p27 peptide	Peptide Targeting Cyclin-binding Groove	CDK2	[375]
Peptide derived from P16^INK4^	Peptide Targeting Cyclin-binding Groove	CDK4, CDK6	[376,377]
**ATP-Non Competitive Small molecules**			
SU9516	ATP-competitive/3-substituted indolinone	CDK4	[325,326]
Compound 1	Small Molecule	CDK4	[378]
3-ATA: 3-amino thioacridone	Aminoacridines	CDK4	[379]
CPD1—3alpha-amino-5alpha androstane	Small Molecule Non-ATP competitive	CDK5/p35	[380,381]
Allosteric pocket in CDK2/CyclinA/p27	Small Molecule Non-ATP competitive	CDK2/cyclinA/p27	[382]
Chrysin-derivative/compound 69407	Small Molecule Non-ATP competitive Allosteric	CDK2 & CDK4/CDK6	[383]
ZK304709/MTGI/ZK-CDK	ATP-competitive	CDK1, CDK2,CDK4, CDK7, CDK9	[384,385]
Cki-277	ATP-competitive/thiazole urea	CDK1, CDK2	[386]
JNJ-7706621	ATP-competitive/acyl-substitutes triazole diamine	CDK1, CDK2/Aurora kinases	[387,388]
RGB-286199	ATP-competitive/indenopyrazole		
AG-024322	Drug-like	CDK1, CDK2, CDK4	[389]
AZD5438	Drug-like	CDK1, CDK2, CDK9	[390,391]
PHA-848125	Drug like	CDK2	
PHA-793887	Drug like	CDK1, CDK2, CDK5, CDK7, CDK9	
BAY-1000394	Drug like	CDK1, CDK4, CDK9	
CINK4	ATP-competitive/triamino-pyrimidine	CDK4, CDK6	[392]
2-Aminoquinazoline inhibitors	Small molecule	CDK4	[393]
7X	Cyanopyridopyrimidine	CDK4 (ARK5)	[394]
	Small molecule	CDK2, CDK4	[395]

### 3.1. ATP-Competitive Inhibitors—From Natural Sources to SYNTHETIC Analogs

The first sources of CDK/Cyclin inhibitors were natural substances purified from bacteria, fungi, marine sponges and plants, such as olomoucine, staurosporine, butyrolactone, flavopiridol and indirubin [240,290,291,292,293,294,298,299,300,301,302,303,305,306,307,308,309,311,312,313,314,318,321,323,325,326,396,397,398,399,400,401,402,403,404]. Despite their antitumoral efficacy, the mechanism of action of these compounds was initially unknown. However, determination of their molecular nature as purine and pyrimidine analogues and further biochemical and structural studies enabled a better understanding of their inhibitory potential and paved the way for development of a class of ATP-binding pocket CDK inhibitors [319,405,406,407].

6-dimethyl aminopurine was the first CDK1 inhibitor identified with an IC_50_ 120 uM [396] Further derivatives lead to discovery of olomoucine IC_50_ 7 uM, a purine analog with selectivity for CDK1, CDK2, CDK5 and MAPK but not CDK4 or 6 [321,397] Purvalanol B is 1000-fold more efficient than olomoucine for CDK2/cyclin A, which owes its overall efficacy to its membrane permeability (IC_50_ 70 nM) for CDK2/cyclin A [318]. 

Staurosporine (antibiotic AM-2282 or STS) is an alkaloid with a bis-indole structure originally isolated in 1977 from *Streptomyces staurosporeus*, which was shown to inhibit CDK1 and related CDKs [294].

Butyrolactone I was initially identified as a metabolite from *Aspergillus terreus* var. *africans* then isolated from cultured medium of microorganisms screened for inhibition of CDK1/Cyclin B [290]. This inhibitor targets CDK1 and CDK2, not CDK4. Despite its poor permeability, butyrolactone has antitumoral effects against several lung, pancreatic and colon cancer cell lines [291,292,293].

Flavopiridol is a semisynthetic flavonoid, and a synthetic analog of rohitukine, a natural alkaloid isolated from the stem bark of the Indian plant *Dysoxylum binectariferum*, with anti-inflammatory and immuno-modulatory, as well as anticancer properties [298]. Initially identified in a tandem screen of the EGF receptor tyrosine kinase cytotoxicity, flavopiridol was found to be more potent towards CDKs, and further biochemical studies as well as determination of crystal structure of flavopiridol complexed to CDK2 provided insights into this specificity [310]. Also known as alvociclib, this wide spectrum CDK inhibitor targets CDK1, CDK2, CDK4, CDK6, CDK7 and CDK9 and displays antiproliferative efficacy in several solid tumours and sarcomas, as well as in leukaemia, lymphoma and multiple myeloma [299,300,301,302,303,310]. Because it targets CDK9, flavopiridol has been reported to interfere with transcription of certain cell-cycle and survival related genes such as c-myc, which would further explain the anticancer potency of this inhibitor [399]. Moreover, flavopiridol constitutes a potent inhibitor for antiretroviral strategies since its ability to target CDK9/cyclin T1. pTEFb has been shown to block RNA pol II CTD-directed kinase activity and transcriptional activation of HIV [400]. However, despite its encouraging potential, this first generation ATP-competitive inhibitor suffered from significant side-effects. A second generation flavone was therefore developed, P276-00, which exhibits higher antiproliferative activity in various tumour cell lines and has entered clinical trials for cancers overexpressing cyclin D, such as multiple myeloma, mantle cell lymphoma and melanoma [305,306].

Indirubin is a bis-indole and the active constituent of a Chinese antileukaemia medicine, Danggui Longhui Wan, that inhibits cyclin-dependent kinases, in particular CDK1 and CDK5, as well as GSK-3β, and refrains cell proliferation by arresting cells at the G2/M transition [323]. SU9516 is 3-substituted indolinone reported to bind and selectively inhibit CDK2/Cyclin A and CDK2/Cyclin E activities, as well as CDK1/Cyclin B in an ATP-competitive fashion, but CDK4/Cyclin D1 in an ATP-non-competitive fashion, yet with 45 fold reduced potency compared to CDK2/Cyclin A. SU9516 induces apoptosis in colon carcinoma cells and kills human leukemia cells through inhibition of RNA Pol II CTD phosphorylation [325,326].

Roscovitine is a trisubstituted purine initially found to be 10 fold more potent inhibitor of CDK1 than oloumicine [398], and constitutes one of the first CDK inhibitors identified which successfully made it through the drug discovery pipeline to clinical trials. Also known as CYC202 or seliciclib, this purine analog primarily inhibits CDK2 and CDK5, as well as CDK1, CDK7 and CDK9 in several forms of human cancers. Like flavopiridol, roscovitine is a good inhibitor of RNA pol II, and has been reported to inhibit HIV [401]. Several potent derivatives have been synthesized and second generation compound CYC065 was proposed for preclinical trials [311,312,313,314].

Hymenialdisine and fascaplysine are two more recent examples of natural compounds identified from marine sponges as inhibitors of cyclin-dependent kinases and cell cycle progression [307,308].

Meriolins [309] are examples of synthetic hybrids, derived from two natural kinase inhibitors extracted from marine invertebrates, meridianins [402] extracted from south atlantic ascidian *Aplidium meridianum*, and variolins [403,404] extracted from the antartic sponge *Kirkpatrickia variolosa*. Meriolins are 3-2-aminopyrimidine indoles, that compete with ATP binding. Variolin B is in preclinical evaluation for cancer therapeutics, yet meriolins display greater specificity for CDKs than variolin B, especially CDK2 and CDK9 as well as better antiproliferative and proapoptotic features than parental counterparts in human cancer cell lines. Meriolin 3 and variolin B bind CDK2/Cyclin A ATP-pocket with very different orientations. Meriolin 3 prevents phosphorylation of CDK1, CDK4 and CDK9 sites, and potently inhibits tumour growth in mouse xenograft models.

### 3.2. From First to Second Generation ATP-Competitive Inhibitors

The first generation CDK inhibitors were essentially *pan*-specific and suffered certain limitations associated with toxic side-effects, which prompted the development of second generation drugs with a more narrow spectrum of selectivity, offering promises of greater efficacy and reduced side effects [408,409,410,411]. The structures of many if not most of these ATP-competitive CDK inhibitors bound to their target have been elucidated, providing important clues to their mechanism of action and specificity/selectivity profile– the moiety outside the ATP pocket determines specificity [412]. Several ATP competitive CDK inhibitors have entered preclinical studies, and there are currently 16 CDK inhibitors in clinical trials (for review [277,278,284,409,410]). 

Although most of these compounds act through inhibition of cell growth and division, several are also very good inhibitors of transcription, targeting CDK7, CDK9 and CDK10. As such, aside for their potential as anticancer therapeutics, they constitute inhibitors of viral infection (e.g., flavopiridol for HIV) [264,413]. Moreover, many of these compounds have proven extremely potent when administered together with other anticancer agents, such as doxorubicin, cisplatin, HER2 inhibitors or HDAC inhibitor vorinostat. In this respect, combination therapies involving administration of both cytotoxic and antiproliferative drugs are more potent and result in less side-effects [409].

Dinaciclib is a pyrimidine derivative which potently inhibits cyclin-dependent kinases CDK2, CDK5, CDK1 and CDK9 *in vitro* with IC_50_ values in the nanomolar range, and inhibits transcription of apoptotic proteins, as well as growth, migration and colony formation of human pancreatic cancer cells, and of several other human cancers *in vivo*. This drug candidate under clinical trials for haematological and solid malignancies including breast cancer is currently in phase 3 for chronic lymphocytic leukemia (CLL) [331,332,333]. CDKI-73 is another pyrimidine which inhibits CDK9, thereby targeting RNA transcription and translation in ovarian cancer cells and synergizing with fludarabine [335,414]. R547 is a diaminopyrimidine which inhibits CDK1/Cyclin B, CDK2/cyclin E and CDK4/Cyclin D which is being evaluated in advanced solid and haematological tumors [328,329].

AT7519 is a pyrazole derivative and multi CDK inhibitor, which has proven efficient towards several human cancer cell lines, which is administered to patients with advanced solid tumours or refractory non-Hodgkin’s lymphoma, and which is currently in phase 2 trials for multiple myeloma, CLL and MCL [349,350,351,352,415] NU6140 [320], NU2058 and NU6027 [316,317] are second generation purines which inhibit CDKs more potently through formation of additional hydrogen bonds with CDK2.

PD-0183812 and PD-0332991*-*Screening of a chemical compound library lead to identification of [2,3-d]pyridopyrimidines as inhibitors of CDK4. The pyrido[2,3-d]pyrimidin-7-one template provided an effective platform for the inhibition of a broad cross-section of kinases, including CDKs [395] and further modification to include a 2-aminopyridine side chain at the C2-position yielded PD-0183812, a potent and highly selective ATP competitive inhibitor of CDK4 and CDK6 kinase activity, which arrested Rb positive cell lines in G1 [336]. On the basis of its selectivity profile and pharmacokinetic profile, PD-0332991 was further identified as a drug candidate for the treatment of cancer [338]. Indeed, PD-0332991, also called palbociclib is a highly selective inhibitor of CDK4-6/cyclin D that blocks retinoblastoma (Rb) phosphorylation in the low nanomolar range, but does not exhibit any activity against a panel of 36 additional protein kinases. Oral administration of PD-0332991 to mice bearing the Colo-205 human colon carcinoma produces marked tumor regression and a net reduction in tumor burden, associated with downregulation of phospho-Rb and Ki-67 as well as of genes under the transcriptional control of E2F [339]. PD-0332991 has proven efficient in advanced cancer and mantle cell lymphoma, in multiple myeloma in combination with other drugs (bortezomib and dexamethasone) and in estrogen receptor-positive advanced breast cancer in combination with letrozole. PD-0332991 also acts synergistically with tamoxifen and trastuzumab in ER+ and HER2 cell lines, respectively. PD-0332991 is one of three oral ATP-competitive selective inhibitors of CDK4 in clinical trials [343]. PD-0332991 is currently tested for liposarcoma, breast cancer and mantle cell lymphoma in phase II or III. LEE011 [344], and LY2835219 [345] are in phase I for melanoma and breast cancer, respectively.

R03306 is thiazolinone and second generation ATP-competitive inhibitor, selective small-molecule inhibitor reveals critical mitotic functions of human CDK1 [348]. SNS-032 thiazole inhibitor that blocks cell cycle progression and transcription by targeting CDK2, 7, 9 [346,347]. More recently, it was shown to target AML cells and to be highly synergistic with cytarabine [416].

### 3.3. ATP-Noncompetitive Inhibitors

Although competitive inhibition by compounds that target the ATP-binding pocket of CDKs has proven an efficient strategy, many of the compounds brought into clinical trials suffer from poor cellular penetration, bioactivity and side effects. Indeed, one of the major issues associated with ATP-competitive inhibitors is their lack of specificity which leads to important cytotoxic side effects and poor tolerability [410]. The dose administered leads to off-target effects that limit efficacy.

In order to circumvent this issue, and to increase the specificity and selectivity of compounds for their target whilst also attempting to limit the emergence of resistance to drugs, aternative strategies have been explored to inhibit CDK/Cyclin function, by interfering with substrate recognition, targeting essential protein/protein interfaces and/or residues required for structural organization, transitional intermediates or conformational changes, rather than tampering with catalytic activity (for review [417,418,419,420]). Indeed, since the ATP-binding pocket is relatively conserved between different members of the CDK family and more generally within the 518 members of the human kinome, targeting distinctive non-ATP binding pockets or protein interfaces provides a means of addressing this issue. ATP-non-competitive inhibitors comprise peptides, peptidomimetics and small synthetic molecules that may behave as substrate-competitive inhibitors, inhibitors of protein/protein interactions and allosteric inhibitors.

#### 3.3.1. Peptide Inhibitors of Protein/Protein Interactions

Protein/protein interactions (PPIs) are essential for recognition and assembly of macromolecular complexes, and offer highly selective interfaces for targeting strategies in drug discovery [421,422,423,424]. Targeting PPIs constitutes an attractive alternative to targeting pockets, yet requires rational design or screening for compounds that compete with or disrupt a specific and relevant interface [423,424,425,426,427,428]. So far several peptide inhibitors have been designed to target PPIs between CDKs and substrates, between CDKs and Cyclins, between CDK/Cyclins and p21/p27/p107 proteins that target the cyclin-binding groove, and peptides that target a surface pocket of cyclin A that plays a central role in recruitment of substrates to the CDK/Cyclin complex [285,287,288,289]. Peptides constitute interesting first generation inhibitors to compete with PPIs, as they may be tailored to mimick specific and complementary interfaces involved in interactions between partners, and may serve as templates for the design of chemical analogs [287,429,430,431,432]. 

##### 3.3.1.1. Substrate-Competitive Inhibitors of CDK2

**Figure 6:**
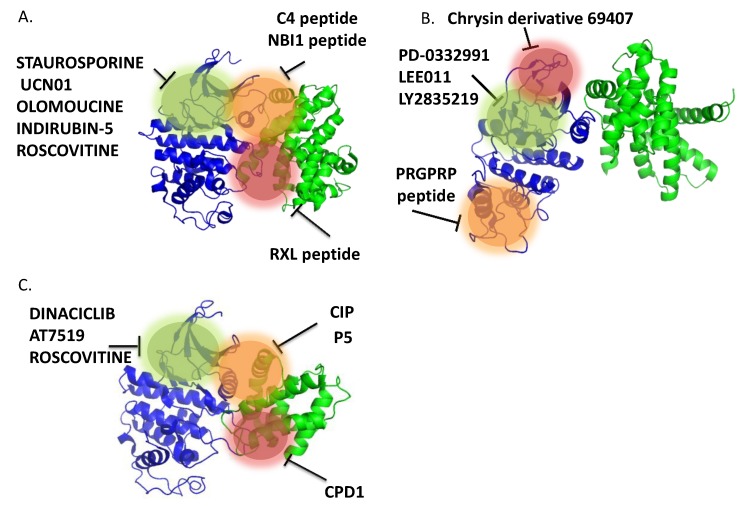
Inhibitors of CDK/Cyclins. (**A**) Structure and inhibitors of CDK2/Cyclin A PDB: 1QMZ; (**B**) Structure and inhibitors of CDK4/Cyclin D PDB 3G33 [CycD3]; (**C**) Structure and inhibitors of CDK5/p25.PDB 1H4L.

A 39mer peptide (Spa310) derived from the RB2/p130 spacer region that mediates the interaction between CDK2/Cyclin A and its RB2/p130 substrate, was found to behave as a competitive inhibitor of substrate binding and CDK2 activity [353]. A 20mer peptide (CIP) derived from a p53 tetramerization domain that binds a CDK2-docking site was reported to efficiently inhibit CDK2 phosphorylation of p53 and induce cell death in melanoma cells [355] (Figure 6A).

##### 3.3.1.2. Peptides Targeting the CDK2/CyclinA Interface

A peptide that targets the primary interface between CDK2 and Cyclin A (PSTAIRE/alpha5 helix) was reported to interfere specifically with CDK2 activity *in vitro* and in cultured cancer cells, although it did not to disrupt CDK2/Cyclin A complex [356]. Another peptide, NBI1, was identified in a screen as binding the surface pocket in cyclin A and inhibiting catalytic activity of CDK2/Cyclin A with high selectivity compared to other protein kinases [357].This peptide binds a structurally conserved domain comprising alpha helices 3, 4 and 5 of cyclin A (involved in the interaction between CDK2 and cyclin A) and competes with cyclin A in disrupting the complex with CDK2 (Figure 6A).

##### 3.3.1.3. Targeting the Cyclin-Binding Groove of CDK2—Mimicking CKIs

The Cyclin Recruitment Motif (CRM) or cyclin-binding groove originally identified by Schulman *et al.* [102] is a conserved hydrophobic motif that lies 35 Ä away from the active site of CDK2 on an accessible alpha helix of cyclin A and serves to recruit several substrates and inhibitors of mammalian CDKs. Adams *et al.* [366] identified a complementary motif in RB1 and RB2 substrates as well as in p21/p27/p57 CKIs which constitutes the binding site with CDK2, and showed that a peptide derived from the cyclin-binding motif of E2F1 transcription factor (PVKRRLDL) could inhibit CDK2/Cyclin A/E kinase activity [366].

Based on this principle, the inhibitory features of CKIs have been exploited to target CDK2 and two classes of p21-competitive peptides have been developed: Nterminal peptides that inhibit CDK/Cyclin activation and *C*-terminal peptides that interact with PCNA and inhibit replication. These include peptides 15–40 and 58–77 [367], 17–33, 63–77 [368], 141–160 [369,433], and 139–164 [370]. Fusions of these peptides with cell penetrating peptides or genetically-encoded GFP of elastin-like polypeptide (ELP) thought to stabilize the peptide in circulation, inhibited CDK/cyclin activation in cell lines. CDK2/cyclin A-E is the main target of these peptides, apart from 141–160 which can also target CDK4/Cyclin D [369]. Furthermore, a peptide derived from the p21 sequence between residues 152 and 159 (mutation S153A) HAKRRLIP allowed to inhibit CDK/Cyclin phosphorylation of Rb substrate, but had no effect on histone H1 (recruitment of which is independent of the cylin-binding groove) [371]. The p27 RNLFGP motif served as a template for design of a constrained peptidomimetic analogue that binds the recruitment site of cyclin A [375]. Whereas linear peptides were not efficient, cyclic peptides displayed efficient inhibitory potential towards CDK/cyclin activity [376]. A peptide bearing the 84–103 (DAAREGFLATLVVHRAGAR) sequence of p16^INK4a^ that interacts with CDK4 and CDK6 was found to inhibit phosphorylation of RB1 by CDK4/cyclin D1 *in vitro*, and block cell cycle progression when fused to Penetratin or TAT, in several cancer cell lines but not in an RB1-negative cell line [374,377] (Figure 6A,B).

##### 3.3.1.4. CDK4 Targeting Peptide

Very few inhibitors other than ATP-competitive inhibitors have been designed to target CDK4/Cyclin D. Warenius and coworkers targeted CDK4 by designing a hexapeptide derived from a *C*-terminal loop outside the kinase domain of CDK4. Cyclic derivatives of this peptide efficiently inhibited proliferation of several cancer cell lines whilst sparing keratinocytes and fibroblasts [365] (Figure 6B).

##### 3.3.1.5. Peptide Inhibitors of CDK5/p25/p35

First-generation inhibitors of CDK5, such as olomoucine, flavopiridol and roscovitine or AT7519 compete with ATP binding but do not act specifically since they also inhibit other CDKs (and other kinases and enzymes that bind ATP). An alternative strategy was developed to interfere with CDK5 hyperactivity and tau hyperphosphorylation by disrupting the interface between CDK5 and p35/p25 [358,359,360,361]. A first peptide of 125 amino acids residues derived from p35, called CIP specifically inhibited CDK5/p25 [359]. A smaller 24 amino acid derivative, called p5, derived from α5 helix of p25 was further found to display greater inhibitory potential than CIP [361]. More recently this inhibitor was applied to mouse models where it was found to reduce neurodegeneration and prevent Alzheimer’s disease [362,363] (Figure 6C).

#### 3.3.2. Small Molecule ATP-Noncompetitive Inhibitors—Allosteric Inhibitors [423,425,434,435]

Recent efforts in drug discovery have focused on the development of compounds which do not compete with ATP, and allosteric inhibitors that bind sites which are not conserved across the kinome, which are accessible in specific conformations only, for instance compounds that bind inactive kinase conformations and stabilize them, or compounds that prevent conformational transitions to active enzymatic states, and which are therefore expected to exhibit superior selectivity profiles [417,418,420,434,435]. The design of such compounds relies on identification of hydrophobic pockets distinct from the ATP-binding pocket, which are exposed only in inactive intermediates, or of allosteric sites. Alternatively, it requires the development of conformation-sensitive assays for high throughput screens [420,378,379].

##### 3.3.2.1. Small Molecule Inhibitors of CDK4 (Figure 6b)

Compound 1 is a 2-aminopyrimidine analog identified as an ATP-non competitive inhibitor of CDK4 in a high throughput screen, which was further found to inhibit Rb phosphorylation in breast cancer cell lines, thereby offering promising perspectives for development of derivatives targeting this kinase [382].

3-ATA or 3-aminothioacridone is an ATP-non competitive inhibitor of CDK4/Cyclin D1 which proved more efficient in inhibiting proliferation of cancer cell types presenting p16 defects than wildtype p16, yet acts independently of p16 binding to CDK4 , suggesting it does not mimic this CKI. This compound has emerged as a promising therapeutic for T-cell acute lymphocytic leukemia [383].

SU9516 is a 3-substituted indolinone that behaves as an ATP-competitive inhibitor of CDK2 and CDK1 kinases, yet as an ATP-non-competitive inhibitor of CDK4 [323,325] (see above). Based on this finding, it is believed that this compound binds a site in CDK4 which is not conserved in CDK1 and CDK2 (distinct from the ATP pocket).

NSC63002 was identified through an *in silico* screen devised to identify compounds binding a specific pocket present only in the inhibited p27KIP-bound form of CDK2-Cyclin A. This compound targets CDK1, CDK2 and CDK4, promoting cytostatic effects associated with decreased Rb phosphorylation and decreased expression of E2F-dependent genes [380]. Moreover NSC63002 docking onto p27-cyclinA-CDK2 reveals that it binds a pocket which is in close proximity, yet distinct from the ATP-pocket.

Chrysin (5,7-dihydroxyflavone), a natural flavonoid widely distributed in plants, reportedly has chemopreventive properties against various cancers. A chrysin derivative, referred to as compound 69407, was found to be an efficient ATP-noncompetitive inhibitor of CDK2 and CDK4 that binds an allosteric pocket in these kinases [381]. This compound attenuated cell cycle progression of EGF-stimulated cells at the G1 phase and inhibited the G1/S transition, causing loss of retinoblastoma phosphorylation by CDK4/6 and CDK2. It also suppressed anchorage-dependent and -independent growth of A431 human epidermoid carcinoma cells and reduced tumor growth in the A431 mouse xenograft model. This study provides new insights for creating a general pharmacophore model to design and develop novel ATP-noncompetitive agents with chemopreventive or chemotherapeutic potency [381].

##### 3.3.2.2. Small Molecule Inhibitors of CDK5 (Figure 6C)

*In silico* and bioluminescence-based screening strategies have also been designed to identify small molecules that target the interface between CDK5 and p25, providing potential leads, such as 3α-amino-5α-androstane for development of new drugs [436,437].

## 4. Concluding Remarks and Perspectives

Cyclin-dependent kinases play central roles in regulation of cell cycle progression as well as in a wide variety of important physiological processes including transcription and neuronal functions. Their deregulation associated with overexpression, amplification or mutation of the CDK or cyclin subunit has been reported in a wide variety of human cancers. Moreover high expression profiles as well as hyperactivity of these heterodimeric kinases is often associated with poor prognosis in patients. CDK/Cyclins therefore constitute attractive pharmacological targets and have been the focus of numerous studies to develop inhibitors which silence or disrupt kinase hyperactivity in human cancers.

Different strategies have been applied to identify compounds that target and interfere with the activity of these kinases, from purification of active compounds from natural substances and high-throughput screening of combinatorial libraries of small synthetic molecules, to the structure-guided, rational design of inhibitors that target ATP-binding pockets, protein/protein interactions or allosteric patches [284,285,286,287,288,289]. 

Although a wide variety of ATP-competitive compounds have been proposed to inhibit these kinases, and there are several successful examples in clinical trials, including Roscovitine, Dinaciclib and Palbociclib, this class of compounds still faces issues with respect to selectivity—there are still many issues in the development of anticancer inhibitors that target the ATP pocket. Indeed, one of the major issues associated with ATP-competitive inhibitors is their limited specificity which leads to important cytotoxic side effects and poor tolerability, since the dose administered necessary to inhibit the kinase target often induces off-target effects that limit efficacy [337]. This being said, second generation derivatives generated through structure-guided approaches that yield drugs with superior specificity and improved therapeutic index promise to increase efficacy and reduce side effects [343,408,410]. Aside from ATP-competitive compounds alternative strategies have been explored to interfere with pockets and interfaces other than the ATP binding pocket of the CDK, including peptides and small molecules, some of which exhibit potent antiproliferative activity, although none of them has yet made it to the clinic.

The challenge consists in developing drugs which inhibit CDK/Cyclin hyperactivity with high efficiency, specificity and selectivity, whilst eliciting minimal toxic side-effects and emergence of resistance over time. To address this challenge, it is essential to understand the nature of the pathology, of the dysregulation, dysfunction, and to define the molecular features of the target. The first step therefore consists in characterizing the biological relevance and targetability, as well as the mechanistic and structural features of the target as thoroughly as possible, so as to identify a specific pocket or interface within the target of interest. Once this information is available inhibitors can be either screened from libraries or designed on a rational basis to target predefined molecular features.

Biochemical and structural studies of cyclin-dependent kinases have provided insights into their molecular mechanisms of regulation, yielding precious information to design novel classes of inhibitors targeting surface hotspots and pockets other than the ATP-binding pocket, competing with protein/protein interactions or targeting essential conformational changes, transitional intermediates. Indeed, it is thought that these classes of inhibitors will provide a higher level of selectivity than ATP-competitive inhibitors, whilst also minimizing the emergence of resistance. Whilst targeting essential protein/protein interactions constitutes a potentially promising approach in terms of specificity, it remains difficult to identify small molecules which can effectively disrupt protein/protein interfaces [422,423,424,425,426]. However, it remains extremely challenging to design allosteric inhibitors through rational approaches, and their identification relies on the discovery of new allosteric sites within target kinases and calls for new strategies and tools for implementing throughput screens [378,379].

Importantly efforts have to be made in targeting the specific subset of functions which are hyperactivated in human cancers since CDK/Cyclin kinases have multiple functions including “housekeeping functions” in regulating transcription or metabolism, which should remain intact so as to generate clinically relevant cytostatic drugs [408]. Moreover a major hurdle in drug discovery remains the efficient intracellular delivery of inhibitors, so as to enable them to penetrate into the cellular cytoplasm and reach their target in an intact and active conformation. Hence optimization of delivery and targeting strategies may constitute a critical step in the development of efficient therapeutic formulations by improving administration efficiency.

Last but not least, the development of combination therapies involving antiproliferative drugs that target CDK/Cyclin kinases and cytotoxic drugs seems to offer encouraging perspectives [409]. Along the same lines, development of polykinase therapeutics targeting multiple kinase targets in the cancer kinome is likely to offer new perspectives for anticancer drug discovery strategies [438]. 
